# Functional Changes in Muscle Afferent Neurones in an Osteoarthritis Model: Implications for Impaired Proprioceptive Performance

**DOI:** 10.1371/journal.pone.0036854

**Published:** 2012-05-14

**Authors:** Qi Wu, James L. Henry

**Affiliations:** Department of Psychiatry and Behavioural Neurosciences, McMaster University, Hamilton, Ontario, Canada; Emory University, United States of America

## Abstract

**Background:**

Impaired proprioceptive performance is a significant clinical issue for many who suffer osteoarthritis (OA) and is a risk factor for falls and other liabilities. This study was designed to evaluate weight-bearing distribution in a rat model of OA and to determine whether changes also occur in muscle afferent neurones.

**Methodology/Principal Findings:**

Intracellular recordings were made in functionally identified dorsal root ganglion neurones in acute electrophysiological experiments on the anaesthetized animal following measurements of hind limb weight bearing in the incapacitance test. OA rats but not naïve control rats stood with less weight on the ipsilateral hind leg (*P* = 0.02). In the acute electrophysiological experiments that followed weight bearing measurements, action potentials (AP) elicited by electrical stimulation of the dorsal roots differed in OA rats, including longer AP duration (*P* = 0.006), slower rise time (*P* = 0.001) and slower maximum rising rate (*P* = 0.03). Depolarizing intracellular current injection elicited more APs in models than in naïve muscle afferent neurones (*P* = 0.01) indicating greater excitability. Axonal conduction velocity in model animals was slower (*P* = 0.04).

**Conclusions/Significance:**

The present study demonstrates changes in hind limb stance accompanied by changes in the functional properties of muscle afferent neurones in this derangement model of OA. This may provide a possible avenue to explore mechanisms underlying the impaired proprioceptive performance and perhaps other sensory disorders in people with OA.

## Introduction

For many people with lower limb osteoarthritis (OA) loss of proprioceptive performance is a significant clinical issue with potential morbidity due to falls. This deficit potentially impacts negatively on functional ability [1]–[7], as activities of daily living require finely-tuned integration of sensory and motor systems. Beyond limitations on functionality, impaired proprioceptive performance has also been linked to variability in walking speed and pattern [8], increased risk of falls [3], [9], [10], and it has even been suggested that impaired proprioceptive performance may be pathophysiologically related to progression of OA [7], [11]–[13]. In fact, it has been advocated that to promote improved functional outcomes in OA, patients’ rehabilitation strategies should be aimed at improving proprioceptive performance [14], [15], particularly in early OA [4], [10], [16]. This evidence suggests that it may be clinically important to understand and to treat the impaired proprioceptive performance in OA patients.

Unfortunately, little is known about neural mechanisms underlying impaired proprioceptive performance in OA patients [2]–[4], [7], [11], [13], [17]. Proprioceptive performance in various tasks including passive movement detection, joint angle reproduction, standing balance, posture and gait requires the central integration of tactile, proprioceptive, vestibular and visual information [18]. Many classes of mechanoreceptor in muscles, joint capsules, ligaments and covering skin are capable of feeding proprioceptive information to higher centres in the brain, with muscle afferent neurones being the most important contributor [19]. Signals from muscles seem to play an especially important role in motor control and thus proprioceptive performance [20]–[22]. Altered muscle afferent discharge is associated with impaired proprioceptive performance, such as greater repositioning error in the lumbosacral spine [23] and decreased control of posture and balance [24].

Although muscle afferent neurones have been suggested to play a critical role in proprioceptive sense and proprioceptive performance, solid evidence about the functional changes in these neurones in OA is still lacking. In a previous study recording intracellularly from dorsal root ganglion (DRG) neurones *in vivo*, we demonstrated that one month after model induction, when this model displays a full spectrum of histopathological and behavioural features of OA [25], significant changes are observed in the properties of evoked action potentials (AP) in a number of functionally differentiated fast conducting A-fibre mechanoreceptors but not in C- or Aδ-fibre nociceptors [26]. These fast conducting A-fibre mechanoreceptors are comprised of various peripheral afferent neurones, including muscle afferent neurones. Receptive field analysis reveals that afferent neurones innervating regions beyond the affected joint and throughout the entire hind limb are involved. This pattern of the widespread changes in A-fibre afferent neurones resembles the neuropathic type of changes [26]. In view of these features, and as there are signs of impaired proprioceptive performance in this model of OA parallel to those reported by OA patients, for the present study we hypothesized that there are accompanying changes in the proprioceptive signals generated specifically, but not necessarily exclusively, by muscle afferent neurones, applying classification criteria defined by Lawson et al. [27].

## Materials and Methods

All experimental procedures were approved by the McMaster University Animal Review Ethics Board and conform to the Guide to the Care and Use of Laboratory Animals of the Canadian Council of Animal Care, Vols.1 and 2. At the end of the acute electrophysiological experiment each animal was euthanized without recovery by an overdose of the anaesthetic.

### Induction of the Animal Model of OA

Procedures for induction of the animal model of OA have been described previously [28]. Briefly, female Sprague Dawley rats (180–225 g) from Charles River Inc. (St. Constant, QC, Canada) were used. Animals were anaesthetized with a ketamine-based anaesthetic (ketamine, 100 mg/ml; xylazine, 20 mg/ml; and acepromazine, 10 mg/ml). The right medial meniscus was removed, and the right anterior cruciate ligament was cut. After surgery, the animals were given 0.05 ml of the antibiotic Trimel (sulfamethoxazole plus trimethoprim; Novopharm, Toronto, ON, Canada) once per day for 3 consecutive days, and the analgesic buprenorphine hydrochloride (Temgesic, Schering-Plough, Kenilworth, NJ, USA) twice per day for two consecutive days.

### Hind Limb Weight Distribution

Standing differential hind limb weight distribution was measured using an incapacitance tester from Linton Instrumentation (Palgrave Diss, Norfolk, UK) to assess proprioception of the ipsilateral hind leg as suggested by Liu et al. [29]. Tests were conducted in naive and in OA animals at one day before surgery and four weeks after surgery; control animals were run at the same times for the purpose of temporal control. Animals were placed in an angled plexiglas chamber positioned so that each hind paw rested on a separate force plate. The force exerted by each hind limb, measured in grams, was averaged over a 5-s period. Three repeated readings were taken. Animals were allowed to acclimate to the chamber for a period of 5–10 min before any readings were taken. The quantification system described by Pomonis et al. was used [30]. The percent weight on the right leg (ipsilateral to the derangement leg) was calculated using the following formula:

% weight on the ipsilateral leg = [weight on the right leg/(weight on the right leg+weight on the left leg)]×100.

### Experimental Setup for *in vivo* Intracellular Recording

Four weeks after model induction, the animal was anaesthetized at a surgical level using the mixture above. The experimental setup and animal preparation for *in vivo* intracellular recording were modified from what has been reported by another research group [27], [31], [32].

The right jugular vein was cannulated for i.v. infusion of drugs. Rectal temperature was maintained at 37°C by a servo-controlled infrared lamp, and the animal was mechanically ventilated to achieve an end-tidal CO_2_ concentration around 40 mmHg. An initial 1 mg/kg dose of pancuronium (Sandoz, Boucherville, QC, Canada) was given i.v. to eliminate muscle tone. Supplements of pentobarbital (CEVA SANTE ANIMALE, La Ballastière, Libourne, France; 20 mg/kg) were given via the i.v. catheter as needed to maintain a surgical level of anaesthesia. Principles for pentobarbital and pancuronium supplements have been described in detail previously [28]; the effect of pancuronium was allowed to wear off periodically to confirm a surgical level of anaesthesia and pupil diameter, and withdrawal reflexes were monitored. Otherwise, a supplement of pentobarbital and pancuronium (1/3 of the initial dose) was administered every hour.

A laminectomy was performed to expose the L4 dorsal root ganglion (DRG) ipsilateral to the surgical derangement. The animal was suspended in a stereotaxic frame. The exposed spinal cord and DRG were covered with warm paraffin oil to prevent drying. The dorsal root of the L4 DRG was cut to allow a 12–15 mm length for electrical stimulation, and one pair of bipolar platinum stimulating electrodes was placed underneath. L4 DRG was chosen for recording because it is one of the DRG containing the most knee joint afferents [33], and also for the convenience of placing vertebral clamps.

Intracellular recordings were made from somata in the L4 DRG using micropipettes fabricated from filament-containing borosilicate glass tubing filled with 3 M KCl solution, with DC resistance of 40–70 MΩ. The microelectrode was advanced using an EXFO IW-800 micromanipulator (EXFO, Montreal, QC, Canada). Intracellular recording was considered to have accurred when a hyperpolarization of at least −40 mV suddenly occurred and an AP could be evoked by electrical stimulation of the dorsal root. Testing was initiated once a stable resting Vm had been recorded for five minutes or more. Evoked APs were recorded with a Multiclamp 700B amplifier (Molecular Devices, Union City, CA, USA) and digitized on-line via a Digidata 1322A interface (Molecular Devices) with *pClamp 9.2* software (Molecular Devices).

**Figure 1:**
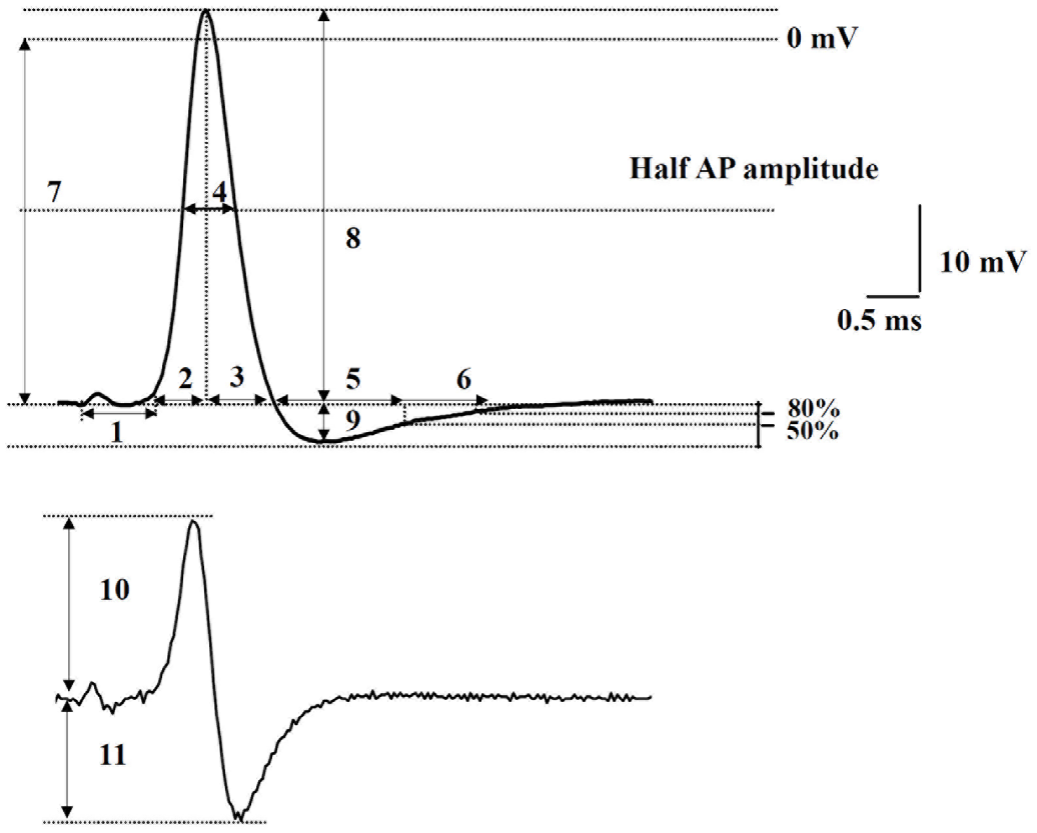
Action potential (AP) recorded intracellularly from a muscle afferent neurone, illustrating parameters measured in each neurone studied. The AP in the upper trace was elicited in a muscle afferent neurone by electrical stimulation of the L4 dorsal root. 1, latency (by measuring the distance from the stimulating site to the centre of DRG after each experiment, the conduction velocity is calculated); 2, AP rise time; 3, AP fall time; AP duration at base (the value equals AP rise time plus AP fall time); 4, AP half width; 5, 50% afterhyperpolarization recovery time; 6, 80% afterhyperpolarization recovery time; 7, resting membrane potential; 8, AP amplitude; 9, afterhyperpolarization amplitude. Lower trace is the differentiated derivative of the upper trace recording, and plots the change of voltage over time: 10, maximum rising rate; 11, maximum falling rate.

Figure 1 illustrates the electrophysiological parameters that were measured in each neurone, including resting membrane potential (resting Vm), action potential duration (APD), AP half width, AP amplitude, AP rise time, AP fall time, maximum rising rate (MRR), maximum falling rate (MFR), afterhyperpolarization (AHP) amplitude, 50% AHP recovery time (AHP50) and 80% AHP recovery time (AHP80). After each experiment the conduction distance was measured for each neurone recorded, as the distance from the centre of the DRG to the stimulation site (cathode). Conduction velocity (CV) was then calculated from this value. Analysis was done offline using the *pClamp 9.2* software.

Once the sensory properties of a neurone had been fully characterized, neuronal excitability was studied. Evaluation of spontaneous activity in muscle spindle neurones was NOT adopted in this study in that it was difficult to accurately differentiate the pathophysiological spontaneous activity due to the increased neuronal excitability and the physiological ongoing discharge resulted from various muscle spindle tensions. The alternative approach to investigate neuronal excitability was to measure the neuronal response to electrical stimulation of the dorsal root as well as to injection of a depolarizing current into the neurone. To measure electrical threshold along the dorsal root, a series of 0.04 ms rectangular pulse stimuli, starting from 0.1 mA with increments of 0.1 mA, were delivered to the dorsal root until an AP could be evoked. The minimum current strength to evoke an AP was recorded as the activation threshold. To determine the response of a neurone to direct current injection, a 20 ms, 2 nA depolarizing current was delivered via the glass pipette. The number of APs occurring during and after current injection was recorded. The measurement of input resistance was unsuccessful due to large variances in value resulted from electrode blocking. This blocking issue was the limitation of the *in vivo* intracellular recording technique in which the recording electrode was very sharp and DRG neurones were still covered by connective tissues and glia.

### Acceptance Criteria

Neurones were included in this study if they exhibited an evoked AP from dorsal root stimulation, had a resting Vm more negative than −40 mV and had AP amplitude larger than 40 mV. For each neurone, before sensory testing was performed a continuous recording was obtained for at least five min after electrode penetration. Only neurones with stable resting Vm throughout recording and sensory testing are included in this report.

### Classification of Muscle Afferent Neurones

Neurones were classified according to established criteria in the published literature. According to the criteria of Harper and Lawson [34] for the classification of CV of peripheral axons in sensory neurones in rats, Aα fibres (an equivalent of Group I fibres) conduct at 30–55 m/s and Aβ fibres (an equivalent of Group II fibres) conduct at 14–30 m/s. Thus, the differentiation CV between Aα fibres and Aβ fibres was set at 18 m/s along the dorsal root (equivalent to 30 m/s along the sciatic nerve according to the report that the dorsal root CV is roughly 0.6 times of the corresponding sciatic nerve value [35]). Moreover, as reported in a previous *in vivo* electrophysiological study in female Wistar rats [36] Aα and Aβ fibre neurones have CVs faster than 6.5 m/s along the dorsal root. We adopted these criteria because they most closely apply to the present study compared to criteria from other labs, as argued in one of our earlier studies [28], including the same gender, a similar age at experiment, similar recording temperature due to a similar surgical exposure, heating strategy and core temperature set-point.

Neurones were also classified as muscle afferents on the basis of the discharge and activation properties defined by Lawson et al. [27], which served as the basis for our present classification criteria. Thus a neurone was classified as a muscle afferent neurone if it could be activated by touching along the muscle belly or changing joint position, and could NOT be activated by touch or pressure stimuli only applied to the covering skin; in this latter case the skin was lifted or pulled aside to ensure that stimuli were not applied to deeper tissue. The neurone must also have a subcutaneous receptive field confirmed by thorough receptive field testing and response to low intensity stimulation of deep structures. Muscles examined for a receptive field encompassed all thigh, calf and toe muscles of the ipsilateral limb.

### Statistical Analysis

Numerical data are presented as mean ± S.E.M. Differences between the weight bearing prior to model induction and four weeks after model induction in the OA group were analyzed with the paired *t*-test. Differences in numbers of neurones between control and OA animals, such as number of APs evoked at specific stimulus strength to the dorsal root or the number of neurones with specific evoked responses following 2nA direct current injection, were analyzed with the Chi-square test. Electrophysiological data were tested for normality using the D'Agostino and Pearson omnibus normality test. Student's *t*-test or the Mann-Whitney *U*-test was used for comparisons between the control and OA model animals, where appropriate. All tests and graphing were done using Prism 4 software from Graphpad (La Jolla, CA, USA). The *P* values of the *t*-tests are indicated in the figures, where appropriate, and *P*<0.05 was set as the level of statistical significance.

## Results

### Effects of Knee Derangement on Hind Limb Weight Distribution

**Figure 2:**
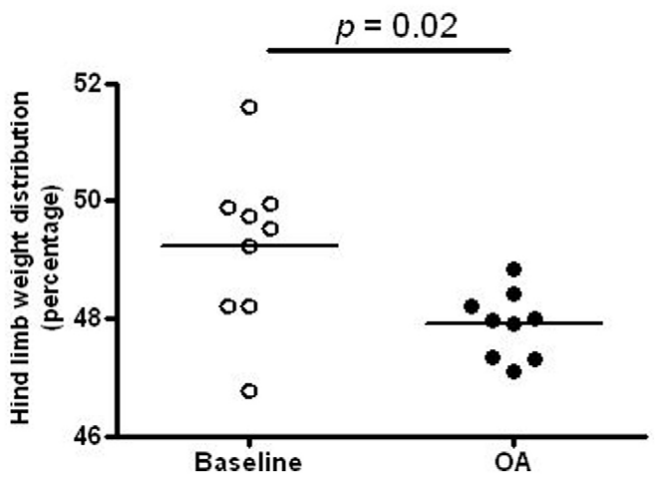
Effects of knee derangement on differential hind limb weight distribution in the incapacitance test. The percentage of weight bearing of the right hind limb (ipsilateral) was compared between one day before surgery (baseline) and 4 weeks after surgery. In each scatter plot, the mean (horizontal line) is superimposed. After confirming that the data was normally distributed, paired *t*-test was used in the comparison. Significant difference in the percentage of weight bearing of the right hind limb between OA and control rats was found at 4 weeks after surgery.

Baseline readings taken before surgery demonstrated equal weight distribution on both hind limbs in both groups of animals and there was no difference between the groups. There was also no difference in the percentage of weight bearing on either leg in control animals after 4 weeks of housing (data not shown). However, at four weeks after surgery in model animals, just before they were used in the acute *in vivo* electrophysiological experiments, 47.9±0.19% of the total hind limb load was placed on the ipsilateral hind limb (*N* = 9). This percentage was significantly less compared to the baseline values in this group before model induction (49.2±0.46%; *N* = 9; paired *t*-test, *P* = 0.02; Figure 2). Before model induction, these model animals almost placed equal weight on both limbs, although slightly more weight either on the left or right hind limb was recorded, but this was randomly distributed. After model induction these model animals consistently placed more weight on the left (contralateral) hind limb. The weight bearing difference between two limbs was significantly increased to 7.5±0.54 gram after knee surgery, which was significant either compared to the baseline value before knee surgery (2.2±1.35 gram, *N* = 9; *P* = 0.002) or compared to the value in a group of control animals with similar age and body weight (1.9±1.46 gram, *N* = 6; *P* = 0.001).

### Electrophysiological Properties of Muscle Afferent Neurones

Successful recordings that met the acceptance criteria were from a total of 35 neurones from 17 control animals and 40 neurones from 14 OA model animals. Among these neurones, 31 out of 35 in control animals and 33 out of 40 in OA animals had CVs faster than 18 m/s, and were thus classified as Aα muscle afferent neurones according to the criteria defined by Lawson et al. [27]. The rest were considered to be Aβ muscle afferent neurones according to these criteria. The slowest conduction velocity measured in these Aβ muscle afferent neurones was 12.3 m/s in control animals, and 12.8 m/s in OA animals. These Aα and Aβ muscle afferent neurones were pooled together in the analysis for the following reasons: 1) we did not have a specific hypothesis regarding the role of different subtypes of muscle afferent neurones in altered proprioceptive functions; 2) there was no evidence of substantial differences in the AP configuration in these two subclasses of muscle afferent neurones, except for an obvious difference in conduction velocity and a difference in the pattern of discharge; 3) even within the same conduction velocity range, muscle afferent neurones never formed a homogenous group, partially because of the variance in innervating structures.

**Figure 3:**
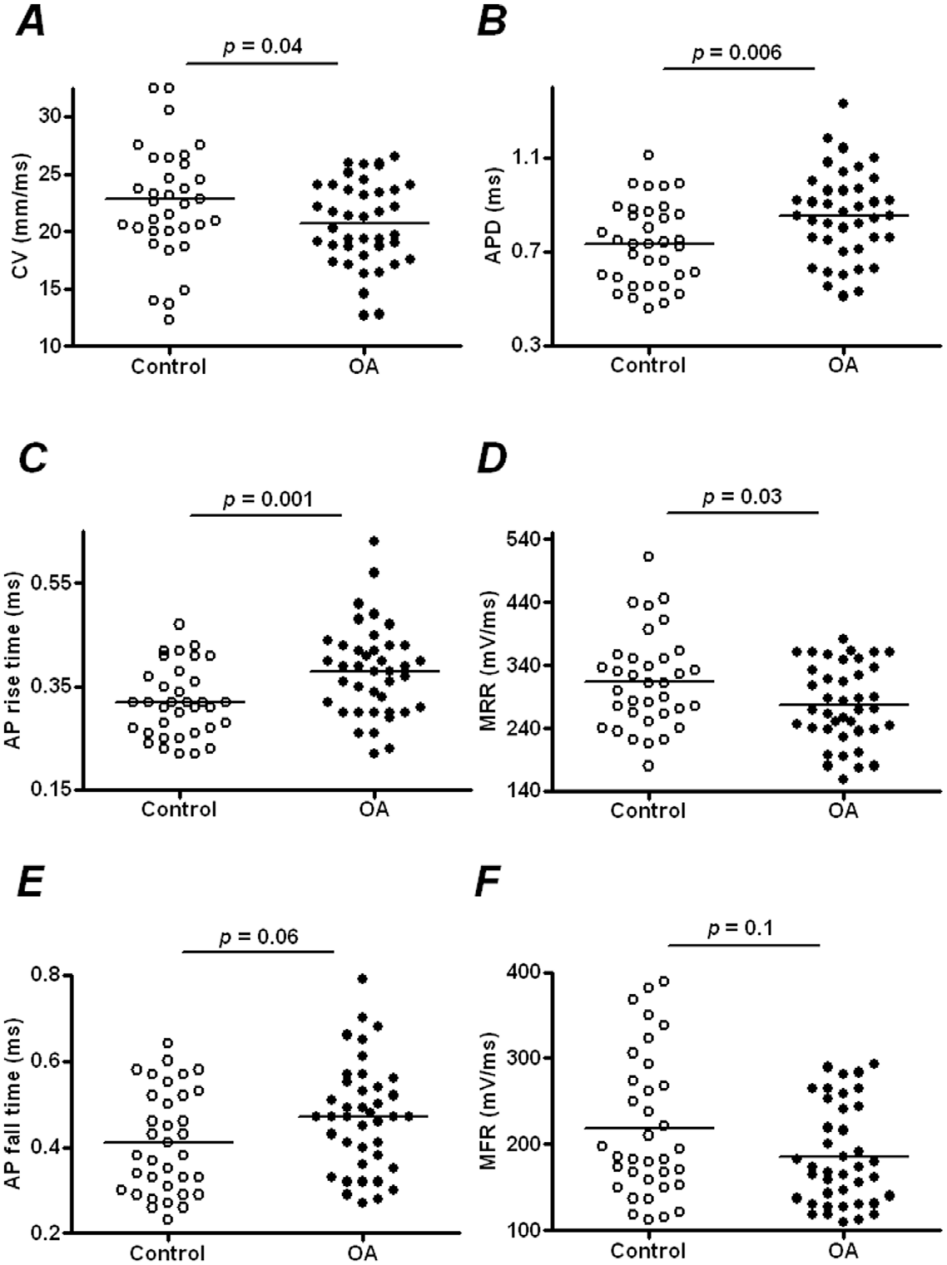
Scatter plots of conduction velocity (A), AP duration (B), AP rise time (C), maximum rising rate (D), fall time (E) and maximum falling rate (F) of individual muscle afferent neurones in control and OA animals. In each case the median (horizontal line) is superimposed. Student’s *t*-tests were used in the comparisons between OA (*N* = 40) and control (*N* = 35) muscle afferent neurones, except that Mann-Whitney *U*-tests were used in the comparison for the AP fall time and maximum falling rate, because the control AP fall time and OA maximum falling rate data failed the D'Agostino and Pearson omnibus normality test. The data indicate slower axonal conduction velocities and slower dynamics of AP generation particularly depolarization in neurones in OA animals.

In control rats, properties of muscle afferent neurones were similar to those in previous *in vivo* reports in guinea-pigs [37], [38], and are also within the range reported from *in vitro* studies [39], [40]. CVs were significantly slower in OA model rats (20.7±0.38 m/s, *N* = 40) compared to control rats (22.9±0.92 m/s, *N = *35; Student's *t*-test, *P = *0.04; Figure 3A).

Resting Vm reflects neuronal excitability, as a depolarized Resting Vm is closer to the threshold of activation of an AP, thereby causing a greater excitability. In all cases, resting Vm remained stable throughout the respective recording session. There was no difference in mean resting Vm between control rats (−61.07±1.21 mV, *N = *35) and OA model rats (−61.65±1.02 mV, *N = *40; Student's *t*-test, *P = *0.72).

APD results from the summation of the depolarizing driving forces and the repolarizing driving forces. Compared with the APD in control rats (0.73±0.03 ms, *N = *35), that in model rats (0.85±0.03 ms, *N = *40) was significantly longer (Student's *t*-test, *P = *0.006; Figure 3B). In addition, another way to evaluate the APD is to measure the width of the AP at half amplitude. AP half width was statistically not different between OA model rats and control rats (0.38±0.01 ms, *N = *40 vs. 0.34±0.01 ms, *N = *35, respectively; Student's *t*-test, *P = *0.06).

The amplitude of an AP results from the summation of the depolarizing driving forces and the repolarizing driving forces of the AP. In the present study, the AP amplitude in muscle afferent neurones seldom exhibited an overshoot, unlike smaller neurones reported by others [38]. There was no difference in AP amplitude between control and OA rats (OA model rats, 54.46±1.15 mV, *N = *40; control rats 55.7±1.55 mV, *N = *35; Student's *t*-test, P = 0.51).

Initiation of an AP is produced by opening of Na^+^ channels, and any change in the type, relative density or opening properties of Na^+^ channels can alter the dynamics of depolarization. AP rise time was taken as the time for depolarization from baseline to peak amplitude. As shown in Figure 3C, there was a longer AP rise time in OA model animals (0.38±0.01 ms, *N = *40) compared to control rats (0.32±0.01 ms, *N = *35; Student's *t*-test, *P = *0.001).

MRR was used as another measure of the dynamics of the depolarization phase of the AP. It was derived by a mathematical conversion of the AP configuration, the derivative of voltage changes during AP with respect to time. Thus, the differentiated curve (Figure 1B) represents the rate of voltage change over time. MRR reflects the maximum depolarization driving force, mostly generated by sodium influx current. Figure 3D shows that MRR was 277.7±9.81 V/s (*N = *40) in the OA rats, which was significantly slower than 313.8±12.63 V/s in control rats (*N = *35; Student's *t*-test, *P = *0.03).

A similar rationale was adopted to determine the dynamics of repolarization, where AP fall time and MFR were used to measure the dynamics of the repolarization phase. Repolarization of the neurone to the resting Vm occurs mainly due to closing of Na^+^ channels and opening of K^+^ channels. This repolarizing phase can influence the rate at which a neurone can discharge. AP fall time had larger individual variance than AP rise time. AP fall time tended to be different from neurone to neurone. This heterogeneity might partly explain the lack of statistical difference revealed in the repolarization phase compared with the depolarization phase. As shown in Figure 3E, no statistical difference in AP fall time was observed in neurones in OA model rats (0.47±0.02 ms, *N = *40) compared to those in control rats (0.41±0.02 ms; *N = *35; Mann-Whitney *U*-test, *P = *0.06).

Similarly, as shown in Figure 3F, MFR was not significantly different in the OA model rats (186.1±9.14 V/s, *N = *40) compared to control rats (218.2±13.89 V/s, *N = *35; Mann-Whitney *U*-test, *P = *0.1).

AHP is generated predominantly by potassium efflux as K^+^ channels close, creating a relatively refractory period. This relatively refractory period governs the maximum rate at which a neurone can discharge. Examination of the parameters of the AHP showed no difference between model and control rats, whether the AHP duration or the AHP amplitude. The AHP amplitude was 8.94±0.67 mV (*N = *39) in OA model rats, similar to the values in control rats (9.4±0.56 mV, *N = *35; Student's *t*-test, *P = *0.61). Furthermore, the AHP50 in OA model rats was similar to that in control rats (1.74±0.14 ms, *N = *39 vs. 1.89±0.16 ms, *N = *35, respectively; Mann-Whitney *U*-test, *P = *0.34), as was the AHP80 (3.42±0.41 ms, *N = *35 vs. 4.13±0.58 ms, *N = *33, respectively; Mann-Whitney *U*-test, *P = *0.29). In 7 neurones (5 in OA rats and 2 control rats), measurements of the AHP associated parameters, particularly AHP80 could not be completed because of greater baseline fluctuation due to a higher noise level during recording.

### Excitability of Muscle Afferent Neurones

Besides measurements of AP characteristics and dynamics of change of membrane potential, neurones were also studied for properties of excitability. Experiments to determine neuronal excitability were done independently of the experiments described above to investigate changes in AP configuration in the somata. A different group of animals was studied to minimize the changes in the neurone properties induced by repetitive discharge. A total of 15 control animals and 17 OA animals were included in this part of the study; 25 neurones from control rats and 37 neurones from OA rats were tested. One experimental protocol tested the activation threshold of dorsal roots; 4 neurones from control animals and 2 neurones from OA animals were not studied.

**Figure 4:**
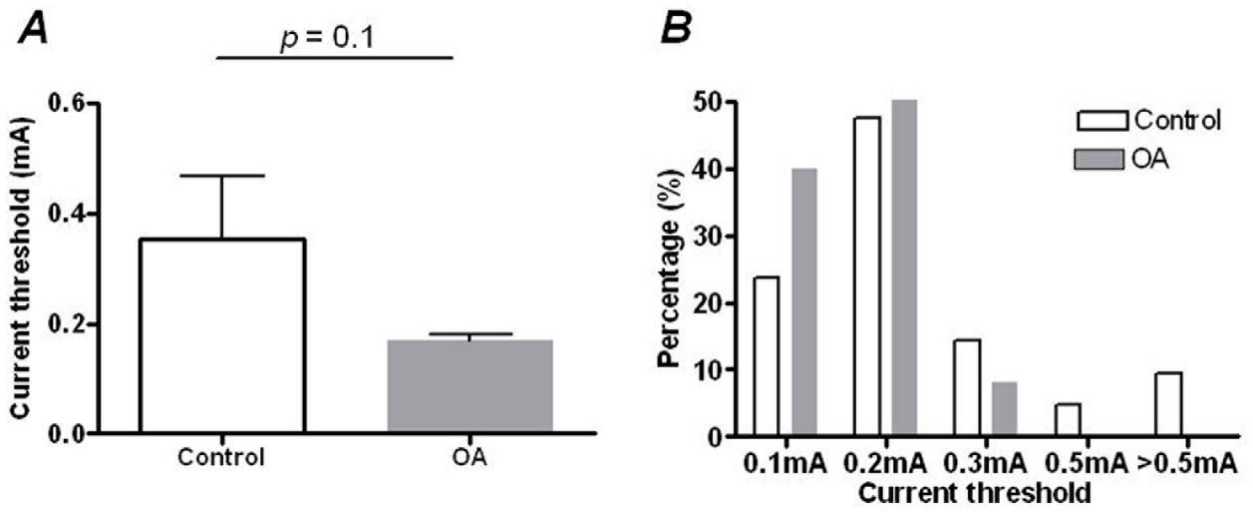
Activation threshold of dorsal root in control and OA animals. A 0.04 ms rectangular pulse stimulus was delivered to dorsal roots at 4 weeks after surgery in control and in OA animals. (A) Shows the comparison of minimal electrical current sufficient to evoke an AP between OA (*N* = 25) and control muscle afferent neurones (*N* = 21). The Mann-Whitney *U*-test was used. (B) Shows the number of neurones evoked at various current strengths to the dorsal root in both control and OA muscle afferent neurones.

To determine the activation threshold of the dorsal root rectangular pulse stimuli were delivered at a current strength just sufficient to evoke an AP. This minimum activating current delivered from the dorsal root in control animals was 0.35±0.12 mA (*N = *21), and was not different from that in OA animals, in which this value was 0.17±0.11 mA (*N = *35; Mann-Whitney *U*-test, *P = *0.1; Figure 4A). In control neurones, the percentage of neurones activated at various current strengths was shown as follows: 0.1 mA (23.8%), 0.2 mA (47.6%), 0.3 mA (14.3%), 0.5 mA (4.8%) and >0.5 mA (9.5%) There was also no difference in the composition of the number of neurones activated at different current strengths between control and OA animals (Chi-square test, *P = *0.29; Figure 4B); in OA animals, the composition was: 0.1 mA (40%), 0.2 mA (52%), 0.3 mA (8%), and none for the 0.5 mA and >0.5 mA current strengths.

**Figure 5:**
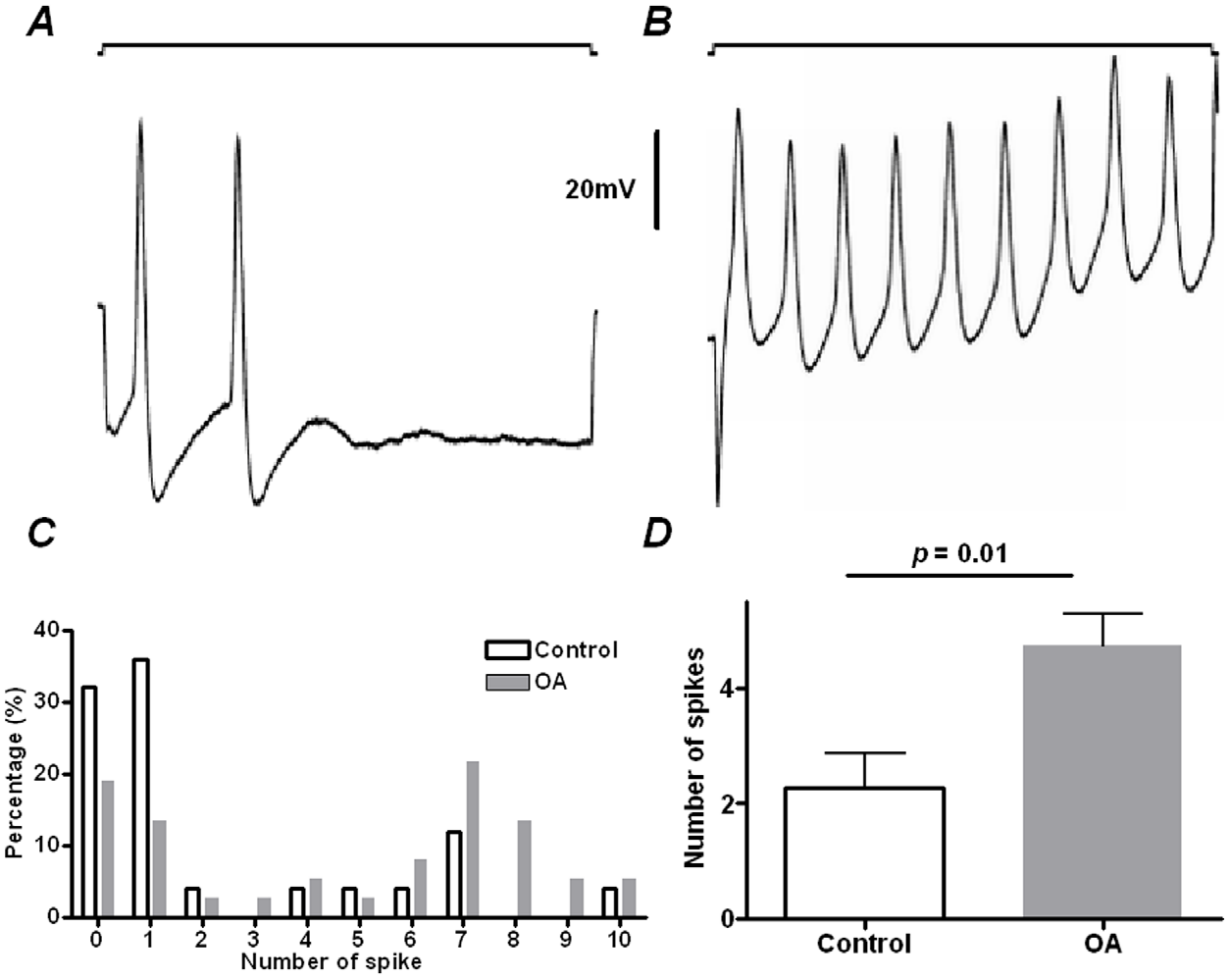
Excitability of muscle afferent neurones determined by depolarizing current injection in control and OA animals. 2nA direct current was injected into neurones at 4 weeks after surgery in control and in OA animals. (**A and B**) show repetitive firing in a control and an OA muscle afferent neurone, respectively. In both recordings, the upper trace indicates the 2 nA depolarizing current, and the lower trace is the intracellular recording signal. (**C**) Shows the histogram showing the number of neurones with various evoked APs following depolarizing current injection in both control and OA muscle afferent neurones. (**D**) Shows the comparison of the number of APs evoked by 2 nA direct current injection between OA (*N* = 37) and control (*N* = 25) muscle afferent neurones. The Mann-Whitney *U*-test was used.

Somata excitability was determined by direct depolarizing current injection into the neurone via the recording pipette. Examples of repetitive firing during direct current injection from muscle afferent neurones in control and OA model animals are shown in Figure 5A, 5B; there was a greater percentage of neurones exhibiting seven or more APs in OA animals: 45.9% in OA (*N = *25) vs. 16.1% in control (*N = *37). In addition, 68% of control muscle afferent neurones exhibited only one AP (36.1%) or no AP (32.2%) following a 20 ms, 2nA depolarizing current injection. In neurones from OA animals this percentage was considerably less, at 32.4%: one AP (18.9%), no AP (13.5%). These differences indicate a significant shift towards greater repetitive firing frequencies during current injection in OA animals (Chi-square test, *P = *0.02). A detailed composition of APs in both groups of neurone is shown in Figure 5C. The average number of APs following the 20 ms, 2nA depolarizing current injection was 2.28±0.59 (*N = *25) in the control muscle afferent neurones. This number was greater in OA muscle afferent neurones, at 4.73±0.57 (*N = *37; Mann-Whitney *U*-test, *P = *0.01; Figure 5D), indicating a greater neuronal excitability in muscle afferent neurones in OA animals.

## Discussion

We present here evidence that there is a change in weight bearing pattern in the surgically-induced knee derangement model of OA, possibly due to a change in posture in OA animals. As we stated previously [26], this model displays histological and imaging profiles resembling those in human knee OA, and mimics the most common aetiology of knee OA [41], which is mechanical injury. Further, we present evidence that there are significant changes in the functional properties of Aα fibre and Aβ fibre neurones innervating the ipsilateral hind limb muscle. These neuronal changes include slower CV, wider APD and slower AP rise time, as well as slower dynamics of depolarization, including slower MRR and an increased number of APs generated during depolarizing current injection. These functional changes in muscle afferent neurones, which thus seem to be correlated with the change in the hind limb weight-bearing pattern in OA animals, might reflect changes in proprioceptive sense and/or proprioceptive reflex, and thus might generate a novel explanation for impaired proprioceptive performance in humans with OA.

### Peripheral Neuropathy and Proprioceptive Performance – an Application in OA

There are two observations in OA model animals highly indicative of neuropathy-like responses that resemble typical changes in models of peripheral neuropathy [39], [42], [43]. These observations include: 1) low-threshold, large myelinated A-fibre neurones underwent functional changes; 2) the nature of AP configurational changes in these neurones, such as broad APD and slow rising/falling rates. These findings are important in understanding OA-related neurological disorders, particularly impaired proprioceptive performance, in that peripheral neuropathy has been shown to be a cause of impaired proprioceptive performance [44]–[48].

The surgical procedure to induce the model involves transection of two highly innervated articular structures, the ACL and the medial meniscus [49], [50]. This might trigger the release of “damage-associated molecules”, such as hyaluronan fragments, fibronectin and myelin debris, and then initiate a cascade of downstream nerve injury-like response, including alternations the expression of various transcription factors and ion channels, via a possible Toll-like receptor mediated mechanism [51].

In the present study, OA muscle afferent neurones were identified innervating thigh, calf and toe muscles, all remote from the initial injury site. This pattern is in line with our previous findings that other low-threshold, large myelinated A-fibre sensory neurones with receptive fields far beyond the knee joint were also altered in function in OA [26], and is also consistent with other evidence in the literature that muscle afferent neurones are commonly affected in various peripheral neuropathies [40], [52], [53].

Altogether, this evidence supports a neuropathy-like functional change in muscle afferent neurones, and further prompts us to suggest that the neuropathy of muscle afferent neurones may also underlie compromised proprioception sense in people with OA. However, further evidence is needed about transcriptional changes in these DRG neurones that reflect nerve injury like responses, such as the expression of ATF3, a cell injury marker in neuropathy models [54]–[56].

It has been reported that patients with various forms of peripheral neuropathy, with diabetic neuropathy being the most studied disease entity, have poor proprioceptive sense, and poor proprioceptive performance including larger body sway and unstable stand [44]–[48]. Importantly, in a study on patients with diabetic peripheral neuropathy postural instability was found to increase linearly with the severity of the neuropathy rather than with the severity of the disease [47].

### Altered Hind Limb Weight Bearing Pattern – Evidence for Impaired Proprioceptive Performance or Nociception or both?

In the present study there was a small yet significant shift in the weight distribution pattern from the ipsilateral to the contralateral hind limb. This raises the question as to how functionally significant this is. Literature review reveals that our observations are in line with previous reports using the gait analysis [57], [58]. In the monosodium iodoacetate-induced OA model, the area/pressure of the ipsilateral paw in contact with the floor, as measured by the CatWalk test, is significant different from that of the control animals [58]. This suggests that these OA animals change the loading on the paw during gait and a change in the weight bearing pattern/posture in the arthritic limb, although the authors attributed these to avoidance of movement of a painful joint [58]. In another study, in the monosodium iodoacetate-induced induced OA model [57], OA animals exhibit clear and consistent reductions in peak vertical load bearing by the affected limb. There is prominent weight bearing redistribution among the four paws, with the contralateral forelimb taking the major share of extra load. Similar results have been reported in this dynamic weight bearing test [59].

Evaluation of proprioceptive performance in human subjects is commonly based on generally adopted tasks, such as passive movement detection and joint angle reproduction [60]. Due to the obvious human-animal difference, these tasks cannot be reproduced in animal studies. Gait analysis and the weight bearing pattern analysis remain two frequently used methods to evaluate gait and or posture in freely moving animals [29], [57], [58]. Afferent discharge in response to externally produced changes of muscle length and tension is another common measurement for proprioception, but in anaesthetized animals.

The incapacitance test, by evaluating the standing/posture might not be a specific measurement of proprioception, as standing/posture is an overall summation of many functions, including muscle strength, skeletal biodynamics, nociception, vision, proprioception, etc. Any difference in weight bearing in this test could be interpreted as a change in posture due to altered proprioceptive performance, or due to a change in joint nociception, or both.

In some studies, differential hind limb weight distribution has been interpreted as an index of joint pain [61], [62]. Even in our recent study in an animal model of bone cancer pain, such a difference, though more robust, was attributed to nociception [63]. Moreover, the incapacitance test has been associated with nociception in inflammatory joint models of OA [30], [61], [62], [64], [65]. Fernihough et al. measured hind paw weight distribution in a surgically-induced rat model of OA and in an inflammatory joint model of OA [66], and reported less robust changes in the surgically-induced model, similar in magnitude to our results.

Here we associate the modest difference in hind limb weight-bearing in OA primarily to stance and proprioception. Riskowski et al. have shown experimentally that the difference in gait kinematics tightly correlates with the difference in the ability to detect motion and reproduce joint angle [67]. In other words, a difference in weight bearing pattern/posture suggests an impaired proprioceptive performance. It is also relevant to point out that in OA animals, there are no overt signs of guarding behaviour that could result in significant changes in standing/posture, including paw lifting and licking and nail pulling or biting, but these signs are commonly observed in various inflammatory and neuropathic chronic pain models [68], [69]. However, this does not preclude the possibility that pain or change in nociception even that is below the level of causing overt guarding responses might partially contribute to a change in weight bearing pattern though unknown nociception-proprioception interaction mechanisms.

However, the interaction of nociception and proprioception might be complex. It is possible that nociceptive signs and proprioceptive alterations are not exclusive to each other. Recently, Felson et al. proposed a change in proprioceptive acuity of knee flexion angle reproduction in OA patients that is associated with the presence and severity of knee pain [2]. Actually, nociceptive and proprioceptive mechanisms influence each other in the spinal cord or in the periphery. Proprioceptive alterations could manifest in patients either without pain or with pain [70]–[73]. In the pain-free population, one should not expect that analgesics would block the proprioceptive alterations. However, in a painful arthritis population, analgesics might improve proprioceptive measurements. For example, in joint angle reproduction task, analgesics might relieve the preventive impacts that a joint pain might have on task performance.

### The Role of Muscle Afferent Neurones in Proprioceptive Performance

We observed significant functional changes in muscle afferent neurones, suggesting a change in muscle sensory input and leading to a possible change in proprioceptive function. In deed, altered posture (static) and gait (dynamic) have been clearly demonstrated in various animal models of OA [57], [58], [61], [66], including our own data. Thus, the purpose of the following discussion is to establish a possible role for altered function of muscle afferent neurons as a contributing mechanism for OA-related neurological disorders, such as gait changes and standing deficits. However, it is important to remain cognizant of the importance of other mechanisms, such as visual and vestibular inputs that contribute to overall stability and proprioceptive performance.

It is widely recognized that sensory information produced by muscle spindles constitutes a crucial part of proprioception [20]–[22]. It has been shown that vibration on muscle tendon induces muscle lengthening and perceived illusory joint movement in the direction that would have stretched the muscle [74]. Group Ia muscle afferent neurones are preferentially activated by vibration on muscle tendon [74]–[76]. It is further reported that sensory input from Group Ia muscle afferent neurones in response to a given movement can be collected, stored, and then translated back into illusory movement via proper vibration on muscle tendon [77], [78].

In the present study, the changes in muscle spindle neurones raise the possibility of altered proprioceptive codes generated from these neurones. Difference in response to somal depolarization, the decreased conduction velocity and the decrease in axonal threshold may be due to common mechanisms, such as changes in membrane resistance or to other factors.

Here we attempt to link the change in posture in OA animals to functional changes in muscle afferent neurones, but other cutaneous or joint mechanoreceptor might also be at play. In our previous studies, it was found that low threshold mechanoreceptors, including cutaneous afferents, Pacinian afferents and glabrous rapid adapting afferents, undergo significant changes in AP configurations [26], which might also play a role in the modulation of proprioceptive reflexes.

Detailed mechanisms of how the neuronal alterations in muscle afferent neurones lead to proprioceptive alterations are still unclear. However, when trying to interpret our data, the following confounding factors should be considered: 1) nociceptive and proprioceptive mechanisms could occur in parallel, and influence each other in the spinal cord, if not also in the periphery; 2) changes in somal APs do not necessarily represent changes in primary afferent axons; 3) distinct ion channel mechanisms might underlie decreased conduction velocity, increased neuronal excitability and slower dynamics of depolarization. Thus, further experiments should be aimed to investigate correlations between neuronal excitability and measurable behaviourally expressed proprioceptive alterations, and to study the role of changes in primary afferent axons in the proprioceptive function.

### Conclusions

We suggest that the impaired proprioception sense associated with OA may be related to neuropathic type of changes in sensory neurones from muscle, based on the similarity in the loss of proprioception sense in people with peripheral neuropathy and those with OA, and the similarity in the neuronal changes in animal models of peripheral neuropathy and the present model of OA.
